# Editorial: The Art of Human-Robot Interaction: Creative Perspectives From Design and the Arts

**DOI:** 10.3389/frobt.2022.910253

**Published:** 2022-06-07

**Authors:** Damith Herath, Elizabeth Jochum, David St-Onge

**Affiliations:** ^1^ Collaborative Robotics Lab, Faculty of Science and Technology, University of Canberra, Bruce, ACT, Australia; ^2^ Research Laboratory for Art and Technology, Aalborg University, Aalborg, Denmark; ^3^ Department of Mechanical Engineering, École de Technologie Supérieure (ÉTS), Montreal, QC, Canada

**Keywords:** human-robot interaction (HRI), human-computer interaction (HCI), social robotics, collaborative robots, robotic art, creative robotics

Advancements in robotics have traditionally been considered the domain of engineering and computer science. However, cross-disciplinary collaborations between the arts and engineering can help drive technical solutions in robotics and fuel innovation in contemporary art (Goldberg, 2001; Herath and Kroos, 2016a; Herath and Kroos, 2016b; Stelarc, 2016). As robotic technologies mature and move beyond research laboratories and the factory floor, there is a greater emphasis and need to understand how to design and implement interactive and collaborative robots in the real world.

User-centred and participatory design methods are well-established in the HCI domain (Wilkinson and De Angeli, 2014), and there is a push to establish similar processes for robot design. Art and design help stimulate this process by involving end-users in the design process and cultivating interdisciplinary approaches for designing and evaluating HRI in the real world. One method for bridging artistic and engineering practices is through workshops that explore diverse disciplinary perspectives to find common ground and identify relevant design principles. Since the early 2010s, many international workshops, forums and programs have explored cross-disciplinary research in robots and art^1^ (Smart et al., 2010; St-Onge, 2019). This research topic expands on ideas and discoveries made by this emerging community.

Human-robot co-creation is an enticing entry point to such exploratory work. Santos et al. explore co-creation by introducing a swarm of robot painters, self-organised and augmenting each other’s limited painting capabilities. The resulting artworks combine the idea of a single artist with a new form of ‘brushstroke’ that emerges from swarm cohesion. The authors show how their control algorithm generates coherent renderings even with varying numbers of robots and painting capabilities. On the other hand, Jansen and Sklar present the findings from a workshop to develop a co-creative drawing system using AI. The work involves a multimodal user study with participants from artistic backgrounds. The authors identify potential pitfalls of introducing robots into the creative process, present a prototype, and discuss the lessons learned through exploring the system's shortcomings.

Carter et al. *Lessons From Joint Improvisation Workshops for Musicians and Robotics Engineers* approaches interaction design by exploring musical improvisation to uncover what robot designers and musicians might learn from one another. The authors identify key themes from human musical improvisation that have relevance for robot designers working on machinic improvisation, including spontaneity, learning, and adaptability. The authors consider how these principles might inform design considerations for robot developers. Jansen and Sklar and Carter et al. also incorporate workshops grounded in participatory design, providing sound strategies for thinking about how to work creatively and productively with stakeholders from diverse backgrounds and expertise.

Mixing designers, artists and engineers into workshop activities is also how Sandry et al. approach their research on communicative social robot design. The authors observe a common practice in designing expressive behaviours for robots is to make robots ‘cute’. However, for many use cases, this may not be desirable. Therefore, the authors propose ‘kawaii’ (Japanese) as a design principle, a concept that encompasses ‘cute’ with playful and surprising personality traits, and provides some valuable insights into the challenges of designing and validating communicative features for social robots.

Gemeinboeck’s *The Aesthetics of Encounter: A Relational-Performative Design Approach to Human-Robot Interaction* and Cuan’s *OUTPUT: Choreographed and Reconfigured Human and Industrial Robot Bodies Across Artistic Modalities* each represent diverse movement-based perspectives for rethinking and reimagining HRI not as we know it but as it might be. Observing how much HRI research is predicated on design strategies that mimic human features and behavior, the authors contrast the anthropomorphic view of robots, instead emphasising the insights that can emerge from exploring interacting with robots along axes of difference. Gemeinboeck proposes a relational-performative approach to designing robots she terms *bodying-thinging,* an approach that expands the possibilities for imagining how robots are designed and how they might participate socially in encounters with people. Gemeinboeck’s work with the Machine Movement Lab (MML) offers alternative pathways that prompt a reconsideration of design for social robots from feminist and movement-based perspectives.

Movement is also the basis for creative inquiry for Cuan’s performance, installation, and augmented reality application *OUTPUT*. Her work considers questions of improvisation, embodiment, and mimesis that are central to automating robot motions and HRI. All three approaches invite people to participate directly and creatively to embody a new relationship with industrial robots through movement. The focus is on investigating the contrasts between mechanical and human bodies, and the different kinds of movements they afford.

Critical perspectives from the arts and humanities, such as gender theory and feminist scholarship, are important concepts for HRI research, especially considering the prevalence of gendered robots and the harmful stereotypes that can find their way into robot design and HRI experimental setups. Ladenheim and LaViers’ *Babyface: Performance and Installation Art Exploring The Feminine Ideal in Gendered Machines* describes an interactive performance installation that promotes critical reflection on gender stereotypes and sexism in robotics. The article reminds readers of the power of art and aesthetic experiences to help open up critical perspectives on assumptions about technology and interaction between people and machines.

LC et al. present a computer vision-based projection and robotic system in an interactive artwork that explores the idea of self-identification, particularly in children. A user study provides additional perspectives and allows the authors to explore the evolving nature of self-awareness in the age of altered human identity through machine interfaces and interventions. Their preliminary findings also point to possible interventions in healthcare, for example by facilitating social interactions for children with communications disorders like autism. On the other hand, Cooney asks the question of which art-making strategy is most appreciated: exogenous, endogenous or hybrid. He introduces the exogenous strategy as following precisely the human input, while the endogenous one does not consider human input. The user study reaches the conclusion that a hybrid solution is preferable to stimulate creativity and motivate the robotic system’s use.

The arts provide an ideal platform for developing robotic solutions and applications that stimulate the public imagination and interest in emerging robot technologies. They also provide a testing ground for studying relevant factors such as intuitive interaction, multimodal communication, trust, design, and usability (Jochum and Goldberg, 2016; St-Onge et al., 2017). The articles in this Research Topic exemplify the kind of novel research outcomes that can result from combining different fields of knowledge.

## Footnotes

1
https://roboticart.org/
